# Reversal of G-Quadruplexes’ Role in Translation Control When Present in the Context of an IRES

**DOI:** 10.3390/biom12020314

**Published:** 2022-02-16

**Authors:** Mohammed Enamul Hoque, Thulasi Mahendran, Soumitra Basu

**Affiliations:** Department of Chemistry and Biochemistry, Kent State University, Kent, OH 44242, USA; mhoque1@kent.edu (M.E.H.); tmahend1@kent.edu (T.M.)

**Keywords:** G-quadruplex, IRES, context dependent, cap-independent translation, cellular mRNAs, ribosome recruitment mechanism

## Abstract

G-quadruplexes (GQs) are secondary nucleic acid structures that play regulatory roles in various cellular processes. G-quadruplex-forming sequences present within the 5′ UTR of mRNAs can function not only as repressors of translation but also as elements required for optimum function. Based upon previous reports, the majority of the 5′ UTR GQ structures inhibit translation, presumably by blocking the ribosome scanning process that is essential for detection of the initiation codon. However, there are certain mRNAs containing GQs that have been identified as positive regulators of translation, as they are needed for translation initiation. While most cellular mRNAs utilize the 5′ cap structure to undergo cap-dependent translation initiation, many rely on cap-independent translation under certain conditions in which the cap-dependent initiation mechanism is not viable or slowed down, for example, during development, under stress and in many diseases. Cap-independent translation mainly occurs via Internal Ribosomal Entry Sites (IRESs) that are located in the 5′ UTR of mRNAs and are equipped with structural features that can recruit the ribosome or other factors to initiate translation without the need for a 5′ cap. In this review, we will focus only on the role of RNA GQs present in the 5′ UTR of mRNAs, where they play a critical role in translation initiation, and discuss the potential mechanism of this phenomenon, which is yet to be fully delineated.

## 1. Introduction

**Figure 1:**
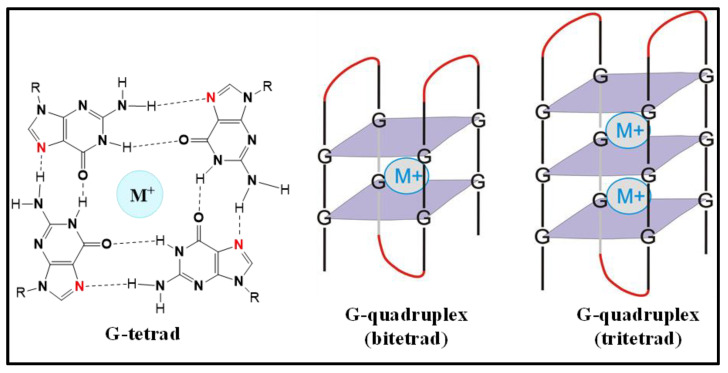
The chemical structure of a guanine tetrad featuring a central metal cation (G-tetrad), two-tier (bi-tetrad) and three-tier (tri-tetrad) G-quadruplexes.

A G-quadruplex (GQ) is a secondary structure adopted by both DNA and RNA, and is formed by the stacking of G-tetrad units. G-tetrads are assembled by the combination of four guanines via the Hoogsteen hydrogen bonds, and the GQ structures are stabilized in the presence of metal cations bound in a plane or in between two tetrads (Figure 1) [1,2,3,4]. It has been found that DNA GQs play regulatory roles in replication, transcription, cell proliferation, genome recombination and telomere maintenance [5,6,7,8,9,10]. On the other hand RNA GQs were found to be involved in translational regulation [11,12,13,14,15,16,17,18,19,20,21,22,23,24], 3′-end processing [25,26], transcription termination [27], alternative splicing [28,29,30,31,32], mRNA localization [33,34,35], protein binding [36,37,38] and telomeric RNA biology [39,40,41,42,43]. Compared with their DNA counterparts, RNA GQs are easier to form, since they do not have to compete with a complementary strand. RNA GQs have been found to be more stable in the folded form, presumably because of the 2′-OH group’s ability to participate in hydrogen bonding [18,44,45,46].

Bioinformatic analysis has revealed more than 4000 GQ motifs in the 5′ UTR (5′ untranslated region) of human mRNAs [47,48,49,50]. The functional consequences of GQ have been experimentally validated in more than 30 5′ UTR sequences from human mRNAs [20,24]. It has been demonstrated that GQ-forming sequences present within the 5′ UTR can function both as vital elements and as repressors of in vitro and in vivo translation [14,18,21,22,51,52]. More interestingly, the role of GQ structures in the modulation of translation depends on the context in which the GQ structure exists [53,54]. It is well known that GQ structures modulate translation in several clinically important mRNAs. Detailed studies of GQ-forming sequences in the 5′ UTR region of various mRNAs, including *NRAS* [18], *ZIC-1* [11], *MT3-MMP* [22] and many others [13,15,16,19,23,24,25,36,37,55,56,57,58,59,60,61,62,63,64], have shown that GQ structures usually serve as repressors of translation and, in most cases, they drive mRNA translation through the cap-dependent mechanism [20,65,66]. The inhibitory effect is often a function of the stability and location of the GQ within the 5′ UTR. The most commonly accepted mechanism of inhibition is GQ-mediated hindrance of ribosomal scanning as it marches toward finding the initiation codon. Other mechanisms of inhibition have also been reported. For example, in case of P1-HNF4A, the two long side chains within the 5′ UTR GQ recruit RNA binding proteins that stabilize the GQ and result in strong repression of translation [67]. In another case, the NRAS GQ was able to inhibit in vitro translation only when it was located in close proximity to the 5′ cap [54], indicating that position matters in the case of the inhibitory effects of the GQ when present within the 5′ UTR of mRNAs. In contrast to the widely reported inhibitory role of the RNA GQs in translation, other reports have demonstrated that the RNA GQs in the 5′ UTR of *FGF2* [14], *VEGF* [21], *ARPC2* [38,68], *TGFβ2* [51], α-synuclein (*SNCA*) [69], *NRF2* [70,71], *BAG-1* [72] and *mTOR* [73] mRNAs are essential for optimal translation. Interestingly, several of these mRNAs harbor Internal Ribosomal Entry Sites (IRESs) that are known to initiate translation in a cap-independent manner. IRES elements are reported to be important during development, stress and many diseases [74,75,76,77,78]. Because IRESs are important in cap-independent translation initiation, GQs embedded in such sequences may play a role in such a process. In fact, in the case of *VEGF* IRES-A, the 40S ribosomal subunit has been shown to interact with the GQ-containing domain [79]. However, in most cases, the function of GQ structures in the IRES-driven cap-independent translation initiation remains to be uncovered Although initially observed in viral mRNAs, IRES-mediated translation initiation has also been identified in many cellular mRNAs [80,81,82]. Generally, cellular IRESs contain fewer RNA structures than viral IRESs and share little sequence conservation among them, making it difficult to classify and predict novel endogenous IRESs in eukaryotic mRNAs. A recent screen using an in vivo translation reporter assay has demonstrated that about 10% of mammalian mRNAs contain certain elements that are involved in IRES function [83]. However, compared with the viral IRESs, the mechanistic aspects of cellular IRES function are poorly understood [66,84,85,86]. Most studies have shown that cap-independent initiation mechanism utilizes IRES elements in case of impaired m7G cap structure recognition at the 5′-end of mRNAs or non-canonical scanning models of translation initiation in eukaryotes [80,85]. However, cap-independent translation initiation can also occur in the absence of an IRES. Some cellular mRNAs use a separate mechanism, known as ‘cap-independent translation enhancer (CITE)-mediated translation’ under apoptotic conditions, which relies on ribosomal scanning of the 5′ UTR [78,87]. Recently, it has also been mentioned that mRNAs containing N6-methyladenosine (m6A) in their 5′ UTR may also be translated cap-independently [88,89,90]. In this mini review, we will primarily concentrate on the role of GQ structures in the context of IRES-mediated cap-independent translation initiation, along with the experimental systems used to investigate them. Related topics such as the regulatory roles of 5′ UTR mRNAs in translation can be found in many recent reviews [20,24,54,91,92], and the biological functions of IRES-mediated cap-independent translation and regulation have also been reviewed recently [65,66,93,94].

## 2. The Role of RNA G-Quadruplexes on IRES-Driven Cap-Independent Translation

**Figure 2:**
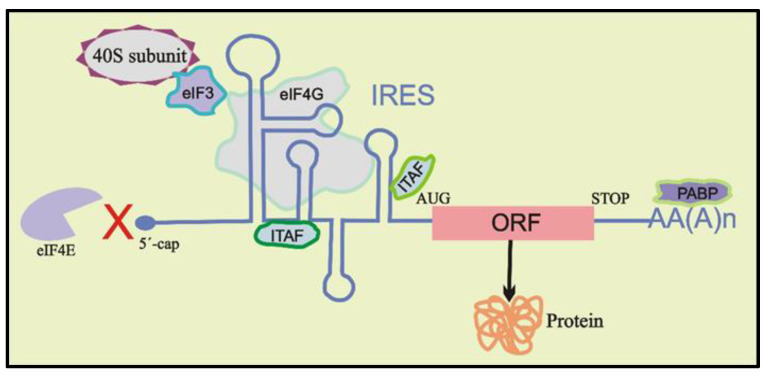
Schematic representation of cap-independent translation. Stem–loop secondary structures within the IRES in 5′ UTR may recruit the 40S ribosomal subunit directly to the start codon (AUG) of the open reading frame (ORF) or its vicinity through direct or indirect interactions, requiring the aid of certain canonical initiation factors (eIFs) and/or IRES trans-acting factors (ITAFs).

The traditional translation initiation mechanism relied on eIF4E binding to the 5′ cap of mRNAs for cap-dependent translation until 1988, when Pelletier and Sonenberg [95] showed that some mRNAs have a mechanism for circumventing the need for eIF4E binding, which was termed IRES-mediated translation initiation. This mode of initiation of translation is usually independent of the identification of 5′ cap structure but may include scanning in a search for the AUG start codon or recruiting the 40S ribosomal subunit directly in the vicinity of the start codon. Recruitment of the 40S subunit may take place either in the complete absence of any other protein factors or with the assistance of specific combinations of canonical initiation factors (such as eIF4G and eIF3) and IRES trans-acting factors (ITAFs) (Figure 2) [96,97]. ITAFs are known to help recruit the 40S ribosomal subunit on the mRNA by different interactions or by stabilizing specific active IRES conformations [81,98,99]. Recently, several studies demonstrated the effect of RNA GQ structures on IRES-mediated cap-independent translation when these structures were located either in the vicinity of the IRES elements or as a part of them [14,21,68,72,73]. Below, we review the known role of GQ structures on IRES activity during cap-independent translation initiation mechanisms.

The role of RNA GQ in the 5′ UTR of mRNAs is mostly described as a repressor of translation, leading to the suggestion that RNA GQ is an inhibitory element for gene expression [17]. However, RNA GQ formation has been shown to promote translation initiation in several cases in a cap-independent manner. Several IRES elements in the 5′ UTRs of cellular mRNAs have been reported to contain GQ motifs. In 2003, Bonnal et al. [14] reported an IRES element within the 5′ UTR of the human fibroblast growth factor 2 (*FGF2*) mRNA from an analysis of the cis-acting elements. They demonstrated that IRES harbors a GQ motif, which is responsible for IRES activity in the translation initiation. They identified the five G-quartets’ RNA GQ structure in the 5′ UTR of *FGF2* mRNA by chemical probing and enzymatic footprinting experiments. Moreover, the mutation and deletion studies revealed that a single 176-nucleotide-long IRES contains two RNA stem–loops and a GQ motif, and the contribution of these structures on IRES activity was able to control translation initiation at four downstream initiation codons within the *FGF2* mRNA. In a recent study, IRES-mediated translation control was demonstrated for the differential expression of FGF9 protein in response to the metastasis of colon cancer cells [100]. Reports indicated that FGF9 protein synthesis is normally low due to upstream open reading frame (uORF)-mediated translational repression; however, it is upregulated in response to hypoxia via an IRES-dependent translational control.

Morris et al. [21] reported that the RNA GQ structure is essential for IRES-mediated translation initiation in the human vascular endothelial growth factor (hVEGF) mRNA. The *hVEGF* mRNA possesses a relatively long (1038 nt) 5′ UTR and harbors two separate IRESs, which are independently capable of initiating cap-independent translation [52,101]. RNase T1 and DMS footprinting assays revealed that a 17-nt (GGAGGAGGGGGAGGAGG) guanine-rich sequence of VEGF IRES-A (293 nt) has a GQ structure. To investigate the roles of GQ on IRESs, we performed a dual-luciferase reporter assay in HeLa cells by introducing mutations in the IRES-A. It was found that the mutation of one of the minimal GQ forming segments had no effect on IRES activity, whereas the quadruple mutant (lacking sufficient Gs to adopt an intramolecular G-quadruplex structure) had lost its activity. We proposed that the double mutant that retained the activity has accommodated an alternate GQ structure by which it can maintain an activity similar to the wild-type. Furthermore, it was proven that the IRES-A forms a switchable, two-tier GQ structure, which is essential for its functional role. The different double mutants analyzed in this study were able to utilize different G-stretches and form alternate quadruplex structures (possible by using different combinations of the five G-stretches) with varying levels of activity. We proposed that the structural flexibility and malleability of the VEGF IRES-A GQ is crucial for its unique function that helps it bind to the factors necessary for translation initiation in a cap-independent manner.

In another study, a subsequent mechanistic analysis by using in vitro RNA footprinting showed that the GQ located in the IRES element of the *VEGF* mRNA interacts directly with the 40S ribosomal subunit in the absence of other protein factors [79]. In 2015, Cammas et al. [102] also analyzed the effect of GQs in *VEGF* mRNA translation. They increased GQ stability by inserting or replacing the sequence to form a 3-G-quartet quadruplex structure and further stabilized it by utilizing GQ ligands. These led to an inhibitory effect on translation, similar to the previously characterized GQs in the 5′ UTR of several mRNAs.

In a recent study, Al-Zeer et al. [68] provided structural and functional evidence that a GQ structure within the 5′ UTR of the actin-related protein 2/3 complex subunit 2 (*ARPC2*) mRNA is essential for IRES-mediated cap-independent translation. The 5′ UTR of *ARPC2* mRNA exists in two variants; the longer variant adopts IRES, which harbors a GQ motif in its central stem-loop element. They investigated the cellular function of the IRES element by measuring the *ARPC2* expression levels as a function of increased cell density-related stress. They observed that the relative *ARPC2* protein level increased with cell density, thus suggesting the expected role of IRES elements in the expression of certain genes that represses cap-dependent translation under stressful conditions [76]. Furthermore, they proposed a model for IRES element folding in the 5′ UTR of the *ARPC2* mRNA based on structural probing experiments and bioinformatic prediction programs. They demonstrated that RNA folds into three hairpins, the middle of which includes an exposed GQ motif on top of the stem–loop structure. Disruption of the GQ structures by site-directed mutagenesis led to an approximately 30% decrease in translational efficiency. The decreased translation efficiency was less pronounced than the value reported previously for the GQ-containing IRES element in the 5′ UTR of VEGF; nevertheless, it showed the functional effect of GQ in IRES-mediated translation initiation [21]. These results indicated that the GQ in the *ARPC2* IRES is at least partly required for its full functionality. In contrast, another study demonstrated that the GQ motif located in the 5′ UTR region of *ARPC2* mRNA plays an inhibitory role in translation. They utilized biophysical characterization for GQ structure confirmation and luciferase reporter assays for translational inhibitory activity, but the exact mechanism of GQ’s roles in the 5′ UTR of *ARPC2* mRNA was not demonstrated [38,103].

Koukouraki and Doxakis [69] reported another IRES element that harbors a GQ motif in the 5′ UTR of the α-synuclein (*SNCA*) mRNA. *SNCA* is a neuronal protein which is likely to be involved in the modulation of synaptic neurotransmission. Several approaches revealed that the 5′ UTR of *SNCA* mRNA has an internal ribosome entry site (IRES) element, which encompasses most of the 5′ UTR. They observed that different cellular conditions, such as depolarization of the plasma membrane, serum malnutrition and oxidative stress, stimulated the translation of α-synuclein protein through its IRES activity. The increased IRES activity not only enhanced expression of a luciferase reporter but also showed a significant increase in endogenous α-synuclein expression [69]. It was shown that the 5′ UTR initiates *SNCA* mRNA translation in a cap-independent process when cap-dependent translation was diminished by rapamycin treatment. The human *SNCA* mRNA has a moderately long 5′ UTR of 264 nt with 66% of GC content that, as expected, has stable stem–loop structures and also includes a GQ motif. To investigate the role of GQ structures in *SNCA* translation or IRES activity, the authors used mutagenized reporter constructs and performed a dual luciferase assay. Functional studies using dual luciferase and real time RT-PCR assays suggested that the GQ in the 5′ UTR of α-synuclein mRNA was not absolutely required for translational activity or IRES functionality, but certainly increased its efficiency. This study serves as another example of GQs’ role in optimal IRES activity.

Recently, Jodoin et al. [72] showed that an RNA GQ located towards the 5′ end of the BAG-1 5′ UTR influences both cap-dependent as well as cap-independent mRNA translation, making it the first report of the involvement of a quadruplex in both mechanisms of translation initiation. In a previous study, the same group identified that the GQ structure within 6 and 35 nt of the BAG-1 5′ UTR repressed the translation of the luciferase gene [63]. They confirmed that this particular quadruplex represses the cap-dependent translation of the main BAG-1 isoform, which agrees with the role of the several previously identified 5′ UTR GQs. Furthermore, they proved that a mutation in the GQ-forming sequence led to the inhibition of cap-independent translation as well, even though it was not present within the IRES. In order to elucidate this phenomenon, they analyzed the effect of deleting the GQ structure in other secondary structural elements in the 5′ UTR, including its IRES, using the selective 2′-hydroxyl acylation analyzed by primer extension (SHAPE) technique. They found that disruption of the GQ structure created base pairing differences mainly in the IRES region of the 5′ UTR, thereby affecting the global folded secondary structure of the 5′ UTR, which, in turn, created the more stable minimal IRES subdomain in the quadruplex mutant compared with the wild-type. On the basis of this observation, they proposed that the structural stability altered because the quadruplex disruption made it more difficult for the IRES to bind to the ITAFs that are essential for 40S subunit recruitment. In conclusion, the study provided an example of a GQ that is essential for maintaining a specific IRES secondary structure to aid cap-independent translation.

Based on a bioinformatics analysis, it has been revealed that transforming growth factor β2 (*TGFβ2*) mRNA has a 23-nt putative GQ-forming sequence in its 5′ UTR. Agarwala et al. performed spectroscopic studies and a luciferase reporter assay to characterize the thermodynamic stability of the GQ and its role in modulating gene expression at the translational level [51]. They found that a construct with the GQ forming sequence together with the flanking sequences, as in the context of the entire 5′ UTR of *TGFβ2*, significantly increased the luciferase expression levels compared with its corresponding mutant. This observation indicated that the GQ within the 5′ UTR of the *TGFβ2* mRNA has a context-dependent effect on translation in which the GQ structure acts as an enhancer of gene expression [51]. The enhancing effect of the GQ structure could be attributed to its location in the 5′ UTR, i.e., it lies further away from both the 5′ end of the UTR and from the translation start site. Unlike the GQ structure in *VEGF* 5′ UTR, which has been previously referred to as a switchable quadruplex that positively regulates cap-independent translation initiation, the *TGFβ2* quadruplex is a stable quadruplex within an unusually long 5′ UTR that positively regulates translation in cap-dependent translation. Although there is an IRES element present in the 5′ UTR of *TGFβ2*, there is no correlation between the role of GQ and IRES functionality. Further study is needed to delineate the precise role of GQ in the context of IRES activity in *TGFβ2* translation initiation.

Human nuclear factor erythroid 2–related factor 2 (*NRF2*) mRNA contains a 555-nt sequence of the 5′ UTR with 70% GC content, which has a 31-nt putative GQ-forming sequence. In 2017, Lee et al. [70] demonstrated the presence of a GQ structure in the 5′ UTR of NRF2 mRNA by utilizing biophysical and biochemical methods, and found that the GQ structure is important for 5′ UTR activity. To identify the exact role of the GQ structure in NRF2 protein translation under oxidative stress, they used proteomics to reveal that elongation factor 1 alpha (EF1a) binds to the GQ sequence. Furthermore, they measured the binding interaction of the EF1a protein with the GQ-forming sequence of 5′ UTR by electrophoretic mobility shift assays (EMSAs) along with RNA–protein interaction assays for cells treated with H_2_O_2_. In addition, their use of small interfering RNA (siRNA) to knock down EF1a by using a reporter assay suggested that the presence of the GQ is important for cellular-level activation of *NRF2* 5′ UTR under oxidative stress. In 2010, Li et al. [104] studied cap-independent translation initiation in NRF2 mRNA via an IRES-mediated mechanism under oxidative stress. They discovered that translation initiation was induced by the binding of 18S rRNA of the ribosome to a highly conserved RNA binding site located within the IRES. According to these two studies, there are two mechanisms through which cap-independent translation is mediated in *NRF2* mRNA, via either the IRES or the GQ. Therefore, it would be interesting to study collectively whether there is any influence of the GQ on IRES-mediated translation initiation, since they both are located in close proximity to each other.

## 3. Mechanism of the Role of G-Quadruplexes in IRES-Mediated Translation Initiation

**Figure 3:**
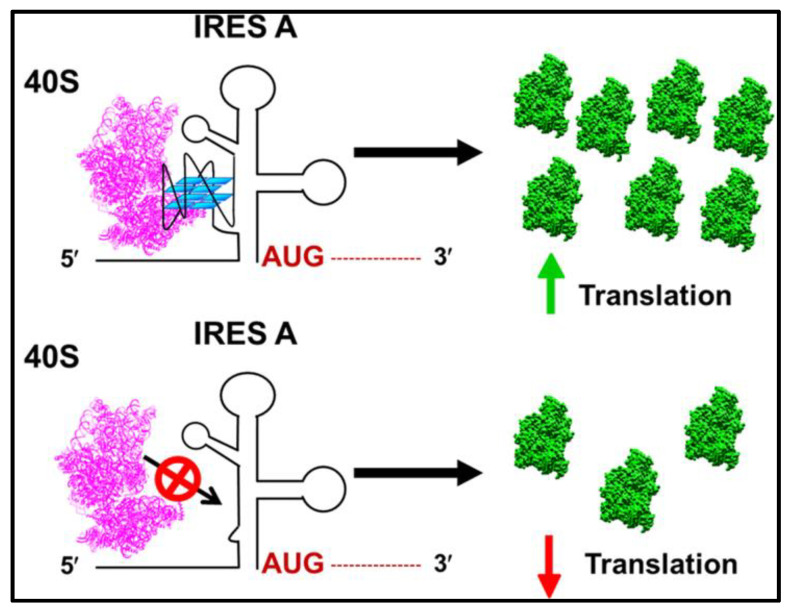
Schematic representation of direct recruitment of the 40S ribosomal subunit by G-quadruplex structures to drive cap-independent translation initiation. Adapted with permission from Bhattacharyya et al., ACS Biochemistry [79].

The initiation of protein synthesis in eukaryotes needs the recruitment of the 40S ribosomal subunit to eventually recognize the start codon (AUG) for translation of the mRNA [105,106,107]. Canonical eukaryotic cap-dependent translation initiation incorporates a complex mechanism for the recruitment and positioning of ribosomes at the start sites, during which, many factors interact with the ribosome [106,108,109]. Alternatively, several viruses and some eukaryotic mRNAs use a cap-independent pathway through highly structured IRES sequences present in the 5′ UTR of mRNAs, which drives pre-initiation complex formation by either positioning the ribosome on or just upstream of the translation start site [66,88,110] (Figure 2). Several studies have shown that GQ structures reside in proximity to the IRES element in 5′ UTR of mRNAs and modulate IRES activity [21,70,71,73,79]. For example, the presence of a switchable GQ structure in the IRES-A of human vascular growth factor (*hVEGF*) mRNA was deemed to be essential for optimum cap-independent translation initiation [21]. To identify the exact role of GQ in IRES-A-mediated translation initiation of *hVEGF*, we utilized structure mapping analyses [79]. Our findings indicated that a 17-nucleotide independently folding RNA GQ domain within the 294-nucleotide IRES-A interacted directly with the 40S ribosomal subunit without the aid of other protein factors (Figure 3). In addition, we showed that the GQ-forming domain particularly dictates the function and binding affinity of IRES-A towards the 40S subunit compared with the bacterial 30S subunit. Further, we proved that GQ domain deletion hindered the 40S subunit from binding to the IRES and deteriorated cap-independent translation initiation, indicating the necessity of the GQ structure in IRES function. Similarly, Marques-Ramos et al. [73] also demonstrated the direct binding interactions of GC-rich secondary structures of *mTOR* 5′ UTR with the 40S ribosomal subunit without the involvement of any initiation factors. They revealed that the *mTOR* 5′ UTR binds to the ribosomal subunit with a similar binding affinity to the CFSV viral IRES by using a filter binding assay.

In another study, indirect evidence of GQ’s role in the binding of IRES to the 40S ribosomal subunit has been investigated [72]. The authors analyzed the effects of GQ structure disruption on other secondary structural elements in the 5′ UTR, including its IRES using SHAPE and found that GQ disruption made it more difficult for the IRES to bind to ITAFs, which is necessary for 40S subunit recruitment. In 2016, Lee et al. [70] reported that a GQ-forming sequence was important for the 5′ UTR activity of NRF2 mRNA through structural mapping experiments [111]. To investigate the exact role of GQ structure in NRF2 protein translation under oxidative stress, they utilized proteomics and EMSA, and revealed the binding interaction of the elongation factor 1 alpha (EF1a) protein with the GQ-forming sequence within the 5′ UTR. Their siRNA-mediated knockdown of EF1α by using a reporter assay suggested that the presence of the GQ is important for NRF2 5′ UTR activation under oxidative stress. Although some studies have demonstrated the direct and indirect role of GQs in IRES activity in the cap-independent translation of various mRNAs, the exact mechanism and potential variations in GQs’ role in ITAF binding in IRES function still need to be adequately deciphered.

## 4. Future Perspectives of G-Quadruplexes’ Effects on IRES-Mediated Translation

G-quadruplexes present within the 5′ UTR modulate translation in both a cap-dependent and cap-independent fashion. IRESs are segments located in 5′ UTR of mRNAs that are capable of recruiting the ribosome and initiating translation independently of the well-understood 5′ cap-dependent initiation mechanism [105,107]. IRESs generally function when 5′ cap-dependent translation initiation has been repressed due to an impaired m^7^G cap structure recognition during development, stress and in many diseases [73,74,79], thereby mediating translation initiation in a cap-independent pathway. There is evidence that the cap-independent mechanism is regulated through RNA secondary structures such as the GQs present within IRESs. However, a limited number of experimentally verified IRES elements have been reported due to the absence of structural and sequence similarities, which make it harder to predict new IRESs in human mRNAs. In 2016, Kwok et al. [112] reported that out of the 3383 RNA GQs observed in the mRNAs, 540 were found in the 5′ UTR. They also identified that in the presence of an rG4 constraint, most of the RNA secondary structures differed extensively, proposing that it not only yields different RNA conformations but can also influence the folding of distal elements. On the basis of these facts and the examples that have been discussed above, we propose that the GQs present within or in the vicinity of an IRES have the potential to either influence IRESs’ functionality or folding [21,51,70,72,104].

Now the question arises as how to predict the putative IRES structures using the mRNA 5′ UTR sequence amid the lack of structural and sequence similarities among the IRESs. Very recently, Tzu-Hsien et al. [113] retrieved 659 human IRESs with experimental evidence based on analyses of databases including IRESite [114,115] and IRESbase [116] together with published literature. Though the number is 659, many of them are from the transcript variants of a particular gene. Combining all the previously identified databases (for example, IRESsite and IRESbase) and available tools, including IRESPred [117], IRESfinder [118] and IRESpy [119], together with the recent reports published by Tzu-Hsien et al. [113] and Bohálová et al. [120], we can find out the experimentally validated IRES elements as well the mRNAs that have the putative IRES-forming sequence in the 5′ UTR. Given the sequences of these selected mRNAs, we can easily predict the presence of GQ sequences within their IRES-forming motifs or in the 5′ UTR in close proximity to the IRES. Currently, GQ detection software such as QGRS Mapper [50], G4 Hunter [121] and QuadBase [122] are available.

We analyzed the 5′ UTR sequences of 659 previously identified human IRESs to check individually whether any of them contained potential GQ-forming sequences. Our analysis using QGRS Mapper revealed that there are 51 5′ UTR sequences corresponding to the mRNAs of 51 different genes that have potential GQ-forming regions with a G score of 20 and above. Among the 51, there are 14 sequences with G scores of above 30 which can form either a three-tiered or a four-tiered GQ, including PTCH1, which had a very high score of 60 (Table 1).

The targets identified using QGRS Mapper were also analyzed using the G4 RNA screener tool in order to find other scores such the G4 Hunter score (G4H), the cGcC score and the neural network score (G4NN). G4 Hunter (G4H) [123] predicts a G quadruplex propensity score based on the G richness and G skewness in a given sequence. G4H uses an optimal threshold score of 0.9 to classify an RNA sequence as a G4 RNA. Bedrat et al. [121] proposed that a G4H score above the threshold of 1.2 would be a good measure for the identification of G4-forming potential in a given sequence, though there will be some false positives and false negatives. All the targets given in Table 1 except two have a G4H score above 1.2. In the case of G4NN, the optimal threshold for G4RNA is 0.5, as it assigns scores between 1 and 0 for G4 and non-G4 RNAs, respectively [124]. Most of our predicted targets have a score that is very close to 1, except for insulin receptor (INSR). The cGcC score is used to evaluate the competition between the formation of GQ and Watson–Crick rule-based structures, as the G stretches involved in the formation of the GQ can base-pair with the nearby C stretches and inhibit GQ formation [125]. The given threshold for the cGcC score in G4 Screener is 4.5. We evaluated the GQ-forming sequences given in Table 1, accompanied by an additional 30 nucleotides upstream and downstream to predict the cGcC score. Beaudoin et al. [125], based on their analysis using 12 potential GQ-forming motifs, suggested that C runs that are present as far as 20 to 50 nucleotides from the GQ motif can influence its folding. They also predicted that candidates above the threshold score of 2.05 can fold into a GQ structure [125]. Among the targets that we analyzed in Table 1, all except FBXW7 and RUNX1 satisfied this particular threshold score.

## 5. Conclusions

The essential role of GQs within a set of 5′ UTR mRNA in upregulating cap-independent translation is still not fully delineated. Unlike the inhibitory effect of GQ within the 5′ UTR, the ones that are involved in the positive regulation are few and far between. G-quadruplexes present in VEGF, alpha synuclein, *ARPC2* and BAG-1 were found to be involved in cap-independent translation that occurs via 5′ UTR IRESs. Although, the abovementioned GQs positively regulate IRES-mediated cap-independent translation initiation, only the GQs of VEGF, alpha synuclein and *ARPC2* are present within the IRES, whereas BAG-1 is located upstream of the IRES. G-quadruplexes in the 5′ UTR of TGFβ2 and NRF2 can also positively influence translation, but their role in the context of IRES is yet to be reported. According to our analysis, the highly structured nature of the IRES is important in modulating cap-independent translation, especially in binding to ITAFs and recruiting the 40S ribosomal subunit. The GQs, when present within or in the vicinity of IRESs, may help display such structural features in order to recruit trans-acting factors. We believe that, considering the context in which GQs are located and their interactions with translation initiation factors as well as ribosomal subunits, experimental analysis of more mRNAs would help us to better understand the role of GQs in IRES-mediated cap-independent translation initiation. Such efforts are poised to be more successful as more IRES databases and GQ prediction tools become available and their findings are coupled with experimental approaches.

## Figures and Tables

**Table 1:** List of mRNAs comprising high G scores (≥30) at the 5′ UTR according to QGRS Mapper.

Name of the mRNAs	Predicted Highest G Score (QGRS Mapper)	cGcC	G4H	G4NN
Leucine zipper protein 6 (LUZP6)	40	6.7778	2.1176	0.9985
Patched 1 (PTCH1)	60	6.0323	2.8000	0.9991
Baculoviral IAP repeat containing 2 (BIRC2)	42	2.6731	1.6333	0.9395
Nuclear factor erythroid 2 like 2 (NFE2L2)	35	1.5571	2.2963	0.9918
MYCN proto-oncogene, bHLH transcription factor (MYCN)	35	4.1154	1.7083	0.9746
Lymphoid enhancer binding factor 1 (LEF1)	36	2.6429	1.4286	0.9449
F-box and WD repeat domain containing 7 (FBXW7)	42	1.1585	1.8889	0.9857
Fibroblast growth factor 2 (FGF2)	41	3.2069	2.3070	0.9962
APC regulator of WNT signaling pathway (APC)	34	3.1556	1.2500	0.9211
Serine hydroxymethyltransferase 1 (SHMT1)	40	3.3871	1.6400	0.9595
MAX network transcriptional repressor (MNT)	38	3.7647	1.5000	0.8408
Insulin receptor (INSR)	34	2.0533	1.0000	0.6700
RUNX family transcription factor 1 (RUNX1)	40	1.1207	1.8333	0.9901
SNF2 histone linker PHD RING helicase (SHPRH)	42	7.3125	2.4000	0.9983

The GQs that were computationally predicted using the available software can be further validated using biophysical and biochemical approaches [126,127] in order to determine further details of the regulatory roles of the GQ structures in IRES-mediated translation initiation.
